# Implications of Homologous Recombination Deficiency for Neoadjuvant Platinum-Based Chemotherapy in Pancreatic Cancer: A Narrative Review

**DOI:** 10.1245/s10434-025-19056-0

**Published:** 2026-01-31

**Authors:** Luigi Liguori, Marco Ventin, Giulia Cattaneo, Liti Zhang, Arsen Osipov, Francesco Sabbatino, Cristina R. Ferrone

**Affiliations:** 1 Department of Surgery, Cedars-Sinai Medical Center, Los Angeles, CA USA; 2 Department of Medicine, Surgery and Dentistry “Scuola Medica Salernitana”, University of Salerno, Salerno, Italy; 3 Department of Medical Oncology, Samuel Oschin Comprehensive Cancer Institute, Cedars-Sinai Medical Center, Los Angeles, CA USA

**Keywords:** BRCA1/2, HRD, Neoadjuvant, PDAC, Platinum

## Abstract

**Background:**

Pancreatic ductal adenocarcinoma (PDAC) remains a highly lethal malignancy, with a 5 year survival rate of approximately 13%. Survival is extended for the few patients who undergo surgical resection with curative intent, whereas most patients succumb to distant disease recurrence. Neoadjuvant platinum-based chemotherapy has emerged as a promising strategy to improve resectability rates and survival outcomes for PDAC patients. However, treatment-related toxicities, unpredictable clinical responses, and associated risk of tumor progression during neoadjuvant therapy may delay or preclude curative resection.As a result, predictive biomarkers are needed to identify patients most likely to benefit from neoadjuvant platinum-based chemotherapy.

**Discussion:**

Alterations in the homologous recombination (HR) DNA repair pathway are reported in 3.0–19.5% of PDAC patients. These types of alterations can sensitize tumors to platinum-based chemotherapy in PDAC as well as other cancers including ovarian, colorectal, and prostate cancers. Retrospective and prospective studies in locally advanced/metastatic PDAC demonstrate higher response rates and longer survival outcomes among HR-deficient (HRD) patients receiving platinum-based chemotherapy. A growing body of evidence in the neoadjuvant setting suggests a potential benefit for HRD-PDAC patients in terms of enhanced tumor downstaging, higher resectability, and improved survival outcomes compared with HR-proficient patients. However, prospective ad hoc studies are still warranted to confirm these findings

**Conclusions:**

Homologous recombination deficiency represents a promising biomarker to guide patient selection for neoadjuvant platinum-based chemotherapy in PDAC. Incorporation of HR deficiency-testing into neoadjuvant treatment schemes will enable a more personalized therapeutic approach, supporting the implementation of precision oncology for early-stage PDAC patients.

Pancreatic ductal adenocarcinoma (PDAC) is among the most aggressive malignancies, with a 5 year survival rate of approximately 13%. Globally, the incidence of PDAC is rising, making it the third leading cause of cancer-related deaths in United States.^1^

The majority of patients present with locally advanced or metastatic disease, and only a limited proportion (10–15%) of patients are eligible for surgical resection at the time of diagnosis.^2^ Even in the case of localized disease, surgery alone is often not sufficient because PDAC is an aggressive systemic disease, and available chemotherapies to date are not sufficiently effective.

The phase 3 PRODIGE24 trial demonstrated improved overall survival (OS) for adjuvant chemotherapy combined with fluorouracil, leucovorin, irinotecan, and oxaliplatin (FOLFIRINOX) compared with gemcitabine for resected PDAC patients.^3^ However, the 5 year disease-free survival (DFS) rate remained suboptimal, at approximately 26%.^4^ In contrast, for patients with locally advanced PDAC who are not eligible for upfront resection, the introduction of induction chemotherapy has improved the survival outcomes, although only a limited proportion of treated patients (<20%) become amenable to radical surgical resection.^5,6^

In the last decade, several studies have demonstrated that neoadjuvant chemotherapy, particularly with cisplatin, oxaliplatin, or carboplatin (platinum-based regimens), improves either resectability or survival outcomes for high-risk resectable, borderline resecatable, and locally advanced PDAC patients compared with upfront surgery.^7–10^ The rationale for a neoadjuvant approach relies on multiple factors, including improved R0 resectability rates and decreased tumor burden, early delivery of systemic therapy to target micrometastatic disease, better tolerance of pre- versus postoperative systemic therapy, and biologic selection. This has prompted evaluation of a neoadjuvant approach even for patients with technically resectable disease. However, a definitive consensus on the use of neoadjuvant chemotherapy versus upfront surgery for resectable PDAC patients has not been reached to date. Two ongoing phase 3 clinical trials (Alliance A021806 and PREOPANC-3) aim to address this clinical question.^11,12^

Despite these advancements, treatment-related toxicities and tumor progression during neoadjuvant chemotherapy can delay or preclude curative resection, posing critical challenges. As a result, predictive biomarkers are needed to identify which patient subgroups most likely benefit from neoadjuvant chemotherapy or upfront surgery. To date, effective and validated biomarkers in this context still are limited.^13^

## Comprehensive Genomic Profiling of PDAC Patients

Comprehensive genomic profiling (CGP) of PDAC patients has been increasingly adopted for patients with PDAC driven by the growing body of evidence supporting experimental use of targeted therapies (e.g., KRAS inhibitors) in advanced stage IV disease. Outside clinical trials, national guidelines do not currently recommend CGP for localized disease until progression occurs after treatment. Although level 1 data supporting this practice at first diagnosis are not available to date, studies have shown that early testing (within 2 months after surgery) improved access to CGP before initiation of post-recurrence treatment, whereas deferred testing was associated with a greater chance of molecularly uninformed treatment.^14^ As our precision surgical oncology knowledge in PDAC expands, early CGP may offer opportunities for tailored treatment as well as improved resectability and survival outcomes at earlier stages of this clinically recognized systemic disease.

## Alterations in the Homologous Recombination Pathway and Their Role in PDAC

The human DNA damage repair (DDR) system comprises many pathways, including base excision repair (BER), nucleotide excision repair (NER), mismatch repair (MMR), homologous recombination (HR), and non-homologous end-joining (NHEJ).^15^ Collectively, these pathways maintain genomic stability integrity by repairing diverse types of DNA lesions such as single-strand breaks, double-strand breaks, base damages, and DNA cross-links.^16^ Dysfunction in one or more of these pathways can lead to genomic instability, thereby increasing the susceptibility of many types of cancers, including PDAC.

**Fig. 1:**
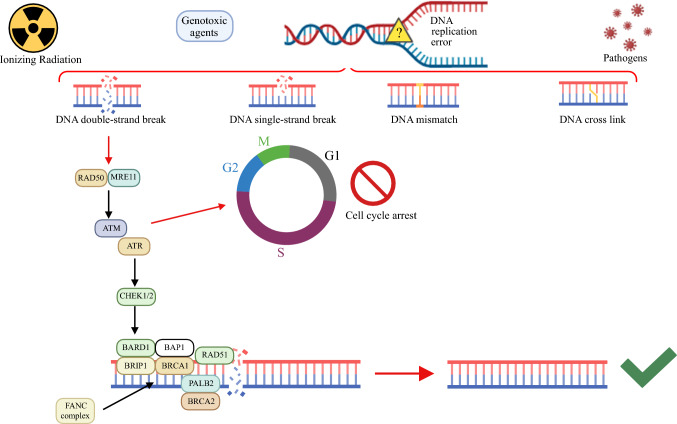
Graphic representation of the key players involved in DNA double-strand breaks via homologous recombination

In the last two decades, significant efforts have been made to elucidate the role of DDR mutations, particularly those affecting the HR pathway, on many types of solid tumors including PDAC. These genetic alterations, present in approximately 3.0–19.5% of PDAC patients^17–19^ include *BRCA1/2* and a large plethora of other genes associated with a “BRCAness” phenotype (Fig. 1).^20^ Collectively, these genes are engaged in the intricate DDR system via the HR pathway.^20^ Notably, alterations in these genes enhance the sensitivity to platinum-based chemotherapy and poly (ADP-ribose) polymerase (PARP) inhibitors in various cancers, including ovarian, colorectal, and prostate cancers.^21–24^ Similar results have been reported in metastatic PDAC patients,^25^ and emerging studies are exploring the impact of HR deficiency in the neoadjuvant setting. In this narrative review, we aim to describe the role of HR deficiency in PDAC, focusing on its potential impact as a predictive biomarker in PDAC patients treated with neoadjuvant platinum-based chemotherapy.

## Prevalence of HR Deficiency in PDAC

Germline HR deficiency is observed in a non-negligible portion of PDAC patients, although reported prevalences vary markedly across studies, reflecting differences in the gene panel size, selection criteria (e.g., family history, early onset), and whether both somatic and germline variants are considered.^17–19,26^ In unselected PDAC cohorts, the most consistently observed alterations involve BRCA2 and ATM. Less frequently, PALB2, BRCA1, and CHEK2 (or Fanconi-pathway genes) are also detected.^17,18,27^ Prevalence data from one of the largest meta-analyses (*n* = 21,482) are summarized in Table 1.^28^ However, gene-level sequencing likely underestimates the true prevalence of HR deficiency. When broader definitions are used (e.g., combining structural variants, genomic scars, and mutational signatures), a substantially larger fraction of PDAC tumors appear to display HRD. Thus, although classical germline/somatic mutations in HR genes provide a lower-bound estimate, comprehensive genomic profiling (including both gene-level and genome-wide readouts) is required to more accurately capture the HR-deficient subpopulation.

**Table 1:** Prevalence of main HR-related gene alterations in PDAC

Gene	Pooled prevalence (germline and somatic)	Pooled prevalence (germline)
*ATM*	2.2%	2.0%
*ATR*	0.1%	–
*BRCA1*	0.9%	0.9%
*BRCA2*	3.5%	3.8%
*CHEK2*	0.3%	0.3%
*FANCA/B/C/D2/E/F/G/I/L*	0.5%	0.4%
*PALB2*	0.2%	0.2%

HR, Homologous recombination; PDAC, Pancreatic ductal adenocarcinoma

## Implications of HR Deficiency for PDAC Biology

Identification of germline HR deficiency can have a significant impact on clinical outcomes for both the cancer patient and healthy carriers. The risk of PDAC development in healthy carriers with germline HR deficiency increases with the number of affected first-degree relatives, reaching 4.6 fold with one affected relative, 6.4 fold with two affected relatives, and up to 32 fold with three or more affected relatives compared with the general population.^29^ Consequently, the National Comprehensive Cancer Network (NCCN) guidelines recommend tailored primary (e.g., smoking cessation and prophylactic surgeries) and secondary (e.g., periodic clinical and imaging surveillance) prevention strategies for all carriers (cancer and non-cancer) based on the specific gene alterations underlying germline HR deficiency.

Alterations in the HR pathway lead to genomic instability resulting in an elevated mutational burden, chromosomal rearrangements, and copy number alterations, all of which can drive tumorigenesis and accelerate tumor progression.^30^ For example, preclinical models have validated the role of BRCA mutations in fostering PDAC progression.^31,32^ However, beyond BRCA, the biologic significance of alterations in BRCAness genes remains underestimated in PDAC to date.

Emerging evidence suggests that homologous recombination-deficient (HRD) PDAC may exhibit a distinct tumor microenvironment (TME). In BRCA-mutated tumors, Shaashua et al.^33^ demonstrated an enrichment of immunoregulatory cancer-associated fibroblasts (CAFs) resulting in heat shock factor 1 (HSF1) upregulation and recruitment of macrophages and T reg cells. In contrast, other studies suggest that mutations in the HR pathway promote a pro-immunogenic phenotype characterized by an increased intra-tumoral CD8+ T cell infiltration and a higher CD8+/FOXP3+ ratio compared with its wild-type counterpart.^34^ Additionally, Seeber et al.^35^ suggested that BRCA1/2 and PALB2 mutations in PDAC are associated with microsatellite instability/deficit mismatch repair (MSI-H/dMMR), elevated programmed death ligand 1 (PD-L1) expression, and a higher tumor mutational burden (TMB) compared with wild-type PDACs. Collectively, these findings hint at a potentially more immunosensitive TME among PDAC patients with HR deficiency despite their overall classification as immunologically cold.^36^ Although, immune checkpoint inhibitor (ICI)-based immunotherapy failed in PDAC, the correlation between BRCA/PALB2 mutations and high TMB^37^ paves the way for testing immunotherapeutic strategies in this subgroup of PDAC patients.

## Therapeutic Implications for PDAC Patients with HR Deficiency

Real-world studies suggest that the adoption of precision medicine can have a substantial impact on the survival of PDAC patients, although molecularly-guided treatments including those targeting oncogenic drivers as well as HR deficiency warrant further prospective evaluation.^38^ Studies have been investigating the potential therapeutic implications of these genetic alterations in both localized and metastatic PDAC disease. The peculiar biologic and immunologic characteristics of HRD-PDAC may influence the sensitivity to personalized oncologic treatments. Specifically, HR deficiency confers high sensitivity to platinum-based chemotherapy and PARP inhibitors by a so-called synthetic lethality mechanism.^39^ The latter is a cell death mechanism triggered by the simultaneous alteration of two genes involved in the DDR system.^39,40^ Accordingly, the simultaneous loss of one DDR gene and PARP inhibition or platinum-induced genetic alterations can trigger cancer cell death.^41^

## Metastatic PDAC

Some retrospective studies have evaluated whether mutations in HR genes influence the outcome for metastatic PDAC patients treated with platinum-based chemotherapy (Table 2). In a retrospective study, after platinum-based chemotherapy, metastatic PDAC patients (*n* = 133) carrying HR mutations were shown to display a better OS than patients without alterations.^26^ Similarly, in a smaller study by Sehdev et al.^42^ metastatic PDAC patients (*n* = 36) carrying HR mutations after treatment with FOLFIRINOX had a longer OS than wild-type patients (median OS, 14 vs 5 months). This benefit was even more significant for patients carrying germline BRCA1/2 mutations. Finally, Park et al.^43^ demonstrated an improved OS for metastatic PDAC patients with HR mutations when treated with first-line platinum-based chemotherapy compared with non-platinum-based regimens. However, they also showed that the survival benefit was observed only for patients carrying either a single mutation in *BRCA1/2* or *PALB2* or a biallelic mutation in the other investigated HR genes.

**Table 2:** Studies investigating the impact of HR mutations in metastatic PDAC patients

Authors	HR mutations	Platinum-based regimen	Line of treatment	Endpoints	Key findings
Goldstein et al.^26^	*ATM*, *BRCA1/2*, *CDKN2A*, *CHEK2*, *ERCC4* and *PALB2*	FOLFIRINOX	First line or subsequent lines	OS	mOS HRD: 18.8 months HRP: 9.1 months
Sehdev et al.^42^	*BRCA1/2*, *PALB2*, *MSH2*, and *FANCF*	FOLFIRINOX	First line	OS	mOS HRD: 14 months HRP: 5 months
Park et al.^48^	*ATM, BAP1, BARD1, BLM, BRCA1/2, BRIP1, CHEK2, FAM175A, FANCA, FANCC, NBN, PALB2, RAD50, RAD51, RAD51C,* and* RTEL1*	FOLFIRINOX FOLFOX Cisplatin plus gemcitabine	First line	OS	mOS HRD: 25.1 months HRP: 15.3 months
Emelyanova et al.^25^	*ATM*, *ATR*, *BARD1*, *BLM*, *BRCA1*, *BRCA2*, *BRIP1*, *CHEK1*, *CHEK2*, *FANCC*, *FANCF*, *FANCG*, *FANCI*, *FANCL*, *FANCM*, *MRE11A*, *NBN*, *PALB2*, *PTEN*, *RAD50*, *RAD51C*, *RAD51D*, *RAD52*, *RAD54B*, *RBBP8*, *RINT1* and *SLX4*	FOLFIRINOX Cisplatin plus gemcitabine	First line	ORR	ORR BRCA/PALB2 mutated: 70% HRD: 22% HRP: 22%

FOLFIRINOX, Fluorouracil, leucovorin, irinotecan, and oxaliplatin; FOLFOX, Fluorouracil, leucovorin, and oxaliplatin; HR, Homologous recombination; HRD, Homologous recombination deficienct; HRP, Homologous recombination proficient; ORR, Objective response rate; OS, Overall survival; mOS, median Overall survival; PDAC, Pancreatic ductal adenocarcinoma

In contrast, an increased susceptibility to platinum-based regimens may not be equally determined by differential alterations across all HR genes because it can be exclusively driven by core mutations in BRCA1/2 or PALB2 genes.^44^ Indeed, Emelyanova et al.^25^ in a cohort of 277 patients with locally advanced/metastatic PDAC, demonstrated a significantly higher percentage in objective response rate (ORR) and survival from first-line platinum regimens only for patients with BRCA/PALB2 germline mutations compared with patients carrying germline mutations in other HR genes as well as in wild-type patients (ORR, 70% vs 22% vs 22%).

Altogether, these findings highlight a major ability of platinum-based regimens to trigger cell death via synthetic lethality for patients with HR deficiency, particularly in those with BRCA/PALB2 mutations. Notably, no significant differences have consistently emerged between the impacts of germline and somatic mutations.

For HRD-PDAC patients, PARP inhibitors have demonstrated some promise. The phase 3 POLO trial demonstrated that maintenance after platinum-based chemotherapy with the PARP inhibitor olaparib significantly improved progression-free survival (PFS), but not OS, for patients with BRCA1/2 mutations.^45^ Similarly, in the phase 2 NCT03140670 clinical trial, treatment with the PARP inhibitor rucaparib of metastatic PDAC patients with BRCA1/2 or PALB2 mutations demonstrated achievement of median PFS and OS values of 13.1 and 23.5 months, respectively, with an ORR of 41.7%, regardless of BRCA1/2 and PALB2 mutations.^46^

The PARP inhibitors are not the only drugs proposed for targeting HRD-PDAC. The specific immunologic phenotype of HRD-PDAC also has been suggested to underlie a higher potential sensitivity to immunotherapeutic strategies. In the phase 2 NCT02693535 clinical trial, administration of ipilimumab (anti-CTLA-4) and nivolumab (anti-PD-1) was associated with low percentages of ORR (14%) and disease control rate (DCR) (31%) in PDAC patients with BRCA1/2 mutations.^47^ Multiple reasons underlie to the limited efficacy of this type of immunotherapy including a high frequency of human leucocyte antigen (HLA) class 1 defects in PDAC.^48^ The latter are well-known to underscore both primary or secondary ICI resistance.^49^ As a result, HLA class 1-independent immunotherapies, such as chimeric antigen receptor (CAR) T cell therapy, are expected to overcome the negative impact of HLA defects in PDAC patients with HR mutations.^50^ Although some clinical trials (e.g., NCT06158139, NCT03323944, NCT03054298) have been investigating CAR T cells in PDAC patients, no ad hoc study in the HRD subgroup has been conducted. Furthermore, combinatorial therapeutic strategies involving PARP inhibitors (e.g., olaparib, niraparib), chemotherapies (both platinum- and non-platinum-based), KRAS inhibitors, and ICIs as well as other types of immunotherapies (lymphocyte-activation gene 3 [LAG3] inhibitors and stimulator of interferon genes [STING\] agonists) are actively under investigation.^51^

## Neoadjuvant Platinum-Based Chemotherapy for HRD-PDAC Patients

Several studies have demonstrated that neoadjuvant platinum-based chemotherapy improves resectability and survival in high-risk resectable, borderline resectable, and locally advanced PDAC compared with upfront surgery.^7,52–54^ However, its role for resectable patients is unresolved, with ongoing clinical trials investigating its utility.^11,12^ Chemotherapy-related toxicity and tumor progression during the neoadjuvant phase can delay or preclude curative resection. Therefore, reliable biomarkers are urgently needed to identify which patient subgroups are most likely to benefit from neoadjuvant chemotherapy or upfront resection.

Given the predictive role of HR mutations in the metastatic setting, recent studies have explored the potential impact of platinum-based therapy in the neoadjuvant setting (Table 3). Pishvaian et al.^55^ retrospectively analyzed the cohort of resected patients enrolled in the “Know Your Tumor” program and categorized PDAC patients as HRD or HR-proficient (HRP) based on the presence of germline or somatic mutations in *BRCA1/2* and *PALB2* (group 1), *ATM*, *ATR*, and *ATRX* (group 2), and *BAP1, BARD1*, *BRIP1*, *CHEK1/2*, *RAD50/51/51B*, or *FANCA/C/D2/E/F/G/L* (group 3). Among resected patients treated with platinum-based chemotherapy, the median OS was 4.5 for the HRD patients, and 2.9 years for the HRP patients. Although it did not reach statistical significance (*P* = 0.08), this difference is clinically relevant for the HRD subgroup. Unfortunately, the small sample of the HRD patients did not allow for further evaluation of the impact of neoadjuvant therapy versus upfront surgery followed by adjuvant chemotherapy. A similar retrospective analysis focusing on *BRCA1/2* and *PALB2* demonstrated a positive trend toward improved OS among HRD patients compared with HRP patients (hazard ratio [HR], 0.15; 95% confidence interval [CI], 0.02–1.23; *P* = 0.07).^56^ Golan et al. ^57^ reported that among borderline resectable PDAC patients treated with neoadjuvant FOLFIRINOX, the pathologic complete response (pCR) rate was 44.4% for the BRCA2-mutated patients and 10% for the BRCA2 wild-type patients (*P* = 0.009). A median DFS was not reached for the BRCA2-mutated patients compared with 7 months for the BRCA2 wild-type patients (*P* = 0.03). Notably, all 8 patients who achieved a pCR were disease-free at the time of analysis. However, given that the study included only patients with BRCA2 mutations, these findings may not be generalizable to other HR mutations.

**Table 3:** Studies investigating the impact of HR mutations in a PDAC neoadjuvant setting

Authors	Study population	Setting	Regimen	HR mutations	Endpoints	Key findings	Main limitations
Pishvaian et al.^55^	PDAC regardless of primary presentation (resected) HRD (*n* = 63) vs HRP (*n* = 314)	Neoadjuvant Adjuvant	FOLFIRINOX Cisplatin with or without gemcitabine	Germline and somatic mutations: Group 1: *BRCA1/2* and *PALB2* Group 2: *ATM*, *ATR*, and *ATRX* Group 3: *BAP1, BARD1*, *BRIP1*, *CHEK1/2*, *RAD50/51/51B*, or *FANCA/C/D2/E/F/G/L*	OS	mOS: HRD: 4.53 years HRP: 2.96 years (*P* = 0.0825)	Retrospective No distinction between neoadjuvant and adjuvant Different chemotherapeutic regimens
Yu, et al.^56^	Non-metastatic PDAC (resected) HRD group (*n* = 32) vs HRP group (*n* = 64)	Neoadjuvant Adjuvant	FOLFIRINOX FOLFOX	Germline mutations: *BRCA1/2* and *PALB2*	OS TTP	mOS: HRD group: not been reached HRP group: 23.1 months (hazard ratio, 0.12; 95% CI, 0.01–1.00) mTTP: HRD group: 13.4 months HRP group: 12.0 months (hazard ratio, 0.74; 95% CI, 0.44–1.25)	Retrospective Small sample size No distinction between neoadjuvant and adjuvant for inclusion Different chemotherapeutic regimens
Golan et al.^57^	Borderline PDAC (resected) BRCA2-mutated group (*n* = 9) vs BRCA2-wild-type group (*n* = 30)	Neoadjuvant	FOLFIRINOX	Germline mutations: *BRCA2*	pCR DFS OS	pCR rate: BRCA2-mutated group: 44.4% BRCA2-wild-type group: 10% (*P* = 0.009) mDFS: BRCA2-mutated group: has not been reached BRCA2-wild-type group: 7 months (*P* = 0.03) mOS: BRCA2-mutated group: has not been reached BRCA2-wild-type group: 32 months (*P* = 0.2)	Retrospective Small sample size Only BRCA2-mutated patients
Ahmed et al.^58^	Resectable, borderline and locally advanced PDAC (resected and not resected) HRD group (*n* = 52) vs HRP group (*n* = 52)	Neoadjuvant	FOLFIRINOX	Germline mutations: *ATM, BARD1, BRCA1, BRCA2, CHEK2, CDKN2A, MLH1, MSH2, MSH3, MSH6, MUTYH, NTHL1, PALB2, PMS2, STK11, TP53*	pCR/near pCR DFS OS	pMR rate: HRD group: 47.2% HRP group: 25.7% (*P* = 0.06) mDFS: HRD group: has not been reached HRP group: 10.8 months (hazard ratio, 0.37; *P* = 0.002). mOS: HRD group: 88.3 months HRP group: 41.3 months (hazard ratio, 0.55; *P* = 0.04).	Retrospective Small sample size

CI, Confidence interval; DFS, Disease-free survival; mDFS, median Disease-free survival; FOLFIRINOX, Fluorouracil, leucovorin, irinotecan, and oxaliplatin; FOLFOX, Fluorouracil, leucovorin, and oxaliplatin; HR, Homologous recombination; HRD, Homologous recombination deficient; HRP, Homologous recombination proficient; OS, Overall survival; mOS, median Overall survival; PDAC, Pancreatic ductal adenocarcinoma; pCR, pathologic Complete response; pMR, pathologic Major response; TTP, Time to progression; mTTP, Median time to progression

A comprehensive bi-institutional analysis by Ahmed et al.^58^ reported data from resectable, borderline resectable, and locally advanced PDAC patients with or without HR mutations (*ATM, BARD1, BRCA1, BRCA2, CHEK2, CDKN2A, MLH1, MSH2, MSH3, MSH6, MUTYH, NTHL1, PALB2, PMS2, STK11, TP53*) treated with neoadjuvant FOLFIRINOX. This study demonstrated a difference in major pathologic response (pCR and near-pCR) from 47.2% for HRD patients compared with 25.7% for HRP patients (*P* = 0.06). The median DFS was not reached for the HRD patients versus 10.8 months for HRP patients (HR, 0.37; *P* = 0.002). The median OS was 88.3 months for the HRP group and 41.3 months for the HRD group (HR, 0.55; *P* = 0.04). In the specific subgroup of resected patients, the median OS was 88.3 months for the HRD patients and 57.1 months for the HRP patients (HR, 0.45; *P* = 0.048), whereas for the specific subgroup of non-resected patients, the median OS was 25.3 months for the HRD patients and 15.2 months for the HRP patients (HR, 0.51; *P* = 0.002).

## Challenges and Future Directions

Despite the promising role of HR deficiency as a predictive biomarker in PDAC, several challenges must be addressed before it can be effectively translated into clinical practice. The first and most critical step is consistent germline and somatic testing for all PDAC patients, as recommended by the NCCN guidelines since 2019.^59^ Unfortunately, not all patients are tested, leading to a missed therapeutic opportunity.^60^ Integration into clinic workflow, education of patients, and accessibility in community and rural settings may represent the keys to improve early testing and integration of genetic data into therapeutic planning.

Although the predictive role of HR alterations, particularly *BRCA1/2*, has been well-validated in metastatic PDAC, current evidence in the neoadjuvant setting is limited. Current studies are retrospective, with fairly small sample sizes distributed across an adjuvant and neoadjuvant approach. Additionally, there is a lack of data regarding the specific subgroups of patients treated with neoadjuvant platinum-based chemotherapy including low-risk resectable, high-risk resectable, borderline resectable, and locally advanced patients. Most studies have demonstrated the predictive role of *BRCA1/2,* but limited data are available on BRCAness genes as well as on the potential differential impact between germline and somatic mutations. Finally, the interpretation of the genotyping results is complicated by the heterogeneity of gene panels used and the molecular biologic testing platforms used.

Future studies are needed to validate the role of different BRCAness gene mutations as predictive biomarkers in the neoadjuvant setting. Addressing these challenges will be crucial to improving the personalized treatment strategy for PDAC patients.
